# Characterization and Applications of Kaolinite Robustly Grafted by an Ionic Liquid with Naphthyl Functionality

**DOI:** 10.3390/ma10091006

**Published:** 2017-08-29

**Authors:** Gustave Kenne Dedzo, Christian Detellier

**Affiliations:** 1 Center for Catalysis Research and Innovation and Department of Chemistry and Biomolecular Sciences, University of Ottawa, Ottawa, ON K1N 6N5, Canada; Christian.Detellier@uottawa.ca; 2 Laboratory of Analytical Chemistry, Faculty of Science, University of Yaoundé I, Yaoundé B.P. 812, Cameroon

**Keywords:** kaolinite, ionic liquid, interlayer grafting, intercalation, nanohybrid material, electroanalysis, adsorption

## Abstract

Functionalization of the kaolinite (K) interlayer space is challenging. In this work, a new kaolinite-based nanohybridmaterial (K-NI) was successfully synthesized by grafting on the interlayer aluminol surfaces the ionic liquid, 1-(1-methylnaphthyl)-3-(2-hydroxyethyl) imidazolium chloride (NI), using a guest displacement strategy. A substantial increase of the basal spacing (10.8 Å) was obtained. This is a grafted derivative of kaolinite possessing one of the largest *d*-values. Washing in water for several days and other vigorous treatments such as sonication showed a minor effect on the integrity of the material. FTIR and ^13^C NMR confirmed the conservation of the structure of the ionic liquid after the grafting. Thermal analysis confirmed the presence of grafted material and was used to estimate the abundance of the grafted ionic liquid (0.44 mole per mole of kaolinite structural formula, (Al_2_Si_2_O_5_(OH)_4_)). By using cyclic voltammetry, the permeability of a film of K-NI for the bulky ferricyanide ions was demonstrated. The accumulation of nitrophenolate anions was effective (maximum capacity of 190 μmol/g), but was less important than what was expected due to the steric hindrance of the bulky grafted NI. Although the presence of chloride anions reduced the adsorption capacity, the affinity of the modified kaolinite interlayer space for the nitrophenolate anions was demonstrated.

## 1. Introduction

Combining the thermal and mechanical stabilities of inorganic materials with the selectivity and specificity of organic groups in organo-inorganic nanohybrid materials results in robust and efficient chemical structures. The reactivity of such nanohybrid materials could also be enhanced by the regular and controlled distribution of organic functionalities on the inorganic support. Mesoporous silica are popular inorganic materials because their structure can be easily tuned to achieve a specific task, and they could be functionalized by various organosilanes after or during their synthesis [1,2,3]. Clay minerals, abundant natural materials, can be used for the same purposes as mesoporous silica, with the advantage of being inexpensive. The 2:1 swelling clay minerals, such as montmorillonite, are the most used: They easily form stable materials with large organic cations intercalated between the layers [4,5]. In addition, silanol or aluminol groups present at their edges, provide reactive sites for the grafting of organosilanes [6,7]. Kaolinite, a 1:1 layered clay mineral, despite its abundance is less used, mostly because of its structure. The individual layer of this clay mineral contains an octahedral aluminum sheet linked to a tetrahedral silicon sheet. Very minor isomorphic substitutions of silicon in the tetrahedral sites, and of aluminum in the octahedral sites, result in a low charge of kaolinite layers. Being asymmetric, these layers have a strong permanent dipole, resulting in the piling of layers, which is further favoured by a dense network of hydrogen bonds between the aluminol and the siloxane surfaces. These two effects strongly maintain the stacking of the layers in the *c* direction. This is at the basis of the book-like structure of kaolinite [8]. Such a structure has a poor chemical reactivity since most of its reactive chemical functions (hydroxyls of the aluminol surface) are non-accessible in the non-swelling interlayer space. However, some salts of small organic anions and a few dipolar molecules such as dimethylsulfoxide (DMSO) or urea can be directly intercalated between the layers of kaolinite with substantial expansion of the interlayer space, and consequently, delamination [9,10]. These pre-intercalates have been widely used as starting material for the intercalation of other compounds (alcohols, amino alcohol, polymers, ionic liquids ...) [11,12,13,14,15,16]. The intercalation of ionic liquids in kaolinite has been demonstrated, and its preparation is well mastered [17]. In several cases, the intercalated ionic liquids were grafted through the formation of an Al–O–C bond [17,18,19]. These kaolinite derivatives, obtained by grafting the cation of the ionic liquid on the internal surfaces of kaolinite have important potential applications. They are in fact organo-inorganic cationic polyelectrolytes, with anion exchanger properties [17,20,21]. In our previous work, it was shown that by controlling the size of the ionic liquid, the d-spacing could be controlled and rigidly fixed, and these materials could then be used as a selective anion sieve [20,21]. Another application is their use as electrode modifiers for anions electroanalysis [22,23,24]. The use of such modified kaolinite to accumulate larger anions necessitates the challenging synthesis of grafted kaolinite derivatives with a larger d-spacing. The grafted kaolinite-based nanohybrid materials resistant to hydrolysis with the largest d-spacing to date have a d_001_ of 15.8 Å [21], and very recently of 17.5 Å [20].

**Scheme 1:**
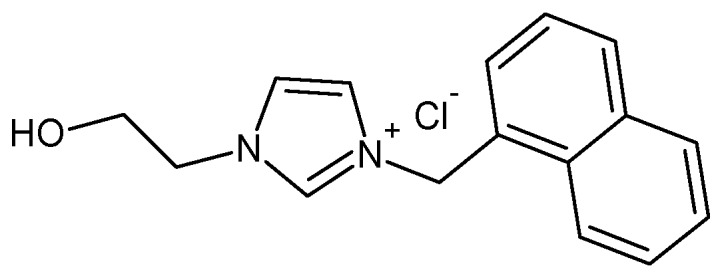
Chemical structure of 1-methylnaphthyl-3-(2-hydroxyethyl) imidazolium chloride (NI).

In this work, a nanohybrid kaolinite-based material was prepared by grafting the ionic liquid 1-(1-methylnaphthyl)-3-(2-hydroxyethyl) imidazolium chloride (NI) (Scheme 1) on the interlayer aluminol surfaces of kaolinite. This material was thoroughly characterized by XRD, TGA, NMR, and Fourier-transform infrared spectroscopy (FTIR). Some applications have subsequently been proposed, including the electroanalysis of ferricyanide, a large anion, and the accumulation of p-nitrophenol (PNP). The affinity of the interlayer spaces for the latter compound was also investigated.

## 2. Results and Discussion

### 2.1. Characterization of the Nanohybrid Kaolinite-Based Material

The characterization of the dimethylsulfoxide pre-intercalate (d_001_ of 11.1 Å with an intercalation ratio of 98%) used as a reaction intermediate is not presented. This kaolinite derivative has been extensively characterized in the literature and the results obtained in this work are in agreement with previously reported results [21,25].

#### 2.1.1. XRD

**Figure 1:**
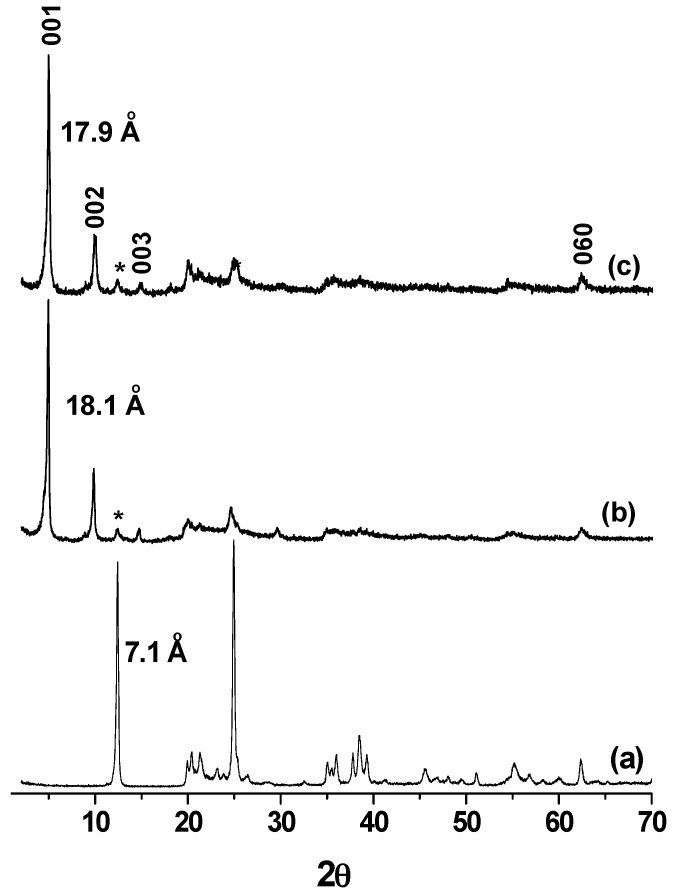
Powdered XRD patterns (diffractometer operating with Cu-Kα radiation (λ = 1.54056 Å)) of (**a**) kaolinite; (**b**) NI intercalated kaolinitewashed with isopropyl alcohol; and (**c**) after washing the product (**b**) in water. Kaolinite residual peaks are marked by *.

The XRD patterns of modified and unmodified kaolinite were recorded in the range 2° to 70°, 2θ. As shown in Figure 1b, the intercalation of the bulky NI in kaolinite results in an expansion along the *c*-direction by increasing the *d*-value from 7.1 Å (for unmodified kaolinite, Figure 1a) up to 18.1 Å. In previous work it was clearly stated that the size and the orientation of the intercalated ionic liquid is related to the expansion of the interlayer [25,26]. The alcohol functional group of the ionic liquid used here can react with the aluminol groups of the internal surfaces to form stable and resistant Al–O–C chemical bonds. To verify the formation of such bonds, the material was dispersed in distilled water, and the suspension was stirred vigorously for 48 h according to the published procedure [27]. The XRD pattern of K-NI in Figure 1c shows a profile similar to that of the material before the treatment with water, with a slight decrease of the d_001_ (from 18.1 Å to 17.9 Å). This decrease results from the de-intercalation of ungrafted fractions of NI [21,27]. The second and the third reflection order (002 and 003) were well defined, indicating the good crystallinity of the material along the c-axis. The residual 001 peak of unmodified kaolinite (marked by *) is attributed to the unreacted fraction of the clay mineral during the DMSO intercalation step. The peak at 62.3° 2θ, corresponding to the d_060_ of kaolinite remained unchanged at different steps of the modification procedure. This indicates that the structural order along the (a,b) plane was maintained.

The stability of K-NI was investigated to ensure the non-variation of the d_001_. Some authors reported the use of ultrasound for exfoliation of kaolinite after being intercalated by bulky compounds [28,29]. In a series of experiments, K-NI was submitted to ultrasounds in distilled water or in toluene for 30 min and the XRD patterns of the recovered solids were recorded (supplementary data, Figure S1). No significant change in the *d*-value was observed, showing that such extreme conditions did not affect the integrity of the material. Despite the large *d*-value of the nanohybrid material, the cohesion between adjacent layers remained important. This suggests strong interactions between the naphtyl group and the silanol surfaces.

#### 2.1.2. TGA

**Figure 2:**
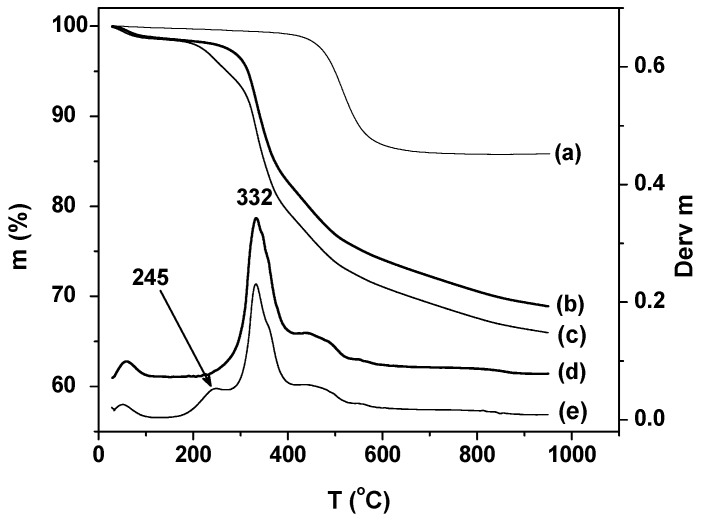
Thermal gravimetric analyse (TGA) curves of (**a**) kaolinite; (**b**) K-NI; (**c**) NI intercalated kaolinitewashed with isopropyl alcohol and DTGA curves of (**d**) K-NI, and (**e**) NI intercalated kaolinitewashed with isopropyl alcohol.

The thermal behavior of the nanohybrid materials was studied by thermal gravimetric analyse (TGA). The thermograms shown in Figure 2 were recorded under identical conditions. Kaolinite has a single mass loss near 505 °C due to dehydroxylation, followed by the formation of metakaolinite. In the case of K-NI, more than one mass loss was observed below 500 °C. The mass loss at 55 °C corresponds to the departure of physisorbed water. The intense peak at 332 °C accounts for the loss of a fragment of NI. Between 400 °C and 490 °C, the broad peak is due to the degradation of residual fragments of NI, and to the dehydroxylation of kaolinite. Above 490 °C, there was a slow and gradual loss of organic matter until the end of the analysis, reflecting the progressive pyrolysis of organic matter trapped in the interlayer space.

The material obtained before washing with distilled water (Figure 2c,e) presents an additional well-defined peak centered at 245 °C, attributed to the loss of ungrafted NI. This observation is in agreement with the interpretation of the decrease of the d_001_ (from 18.1 Å to 17.9 Å) observed after washing with water, which was rationalized as the loss of non-grafted NI.

To determine the amount of grafted material, the TGA of K-NI was recorded under air. This oxidizing atmosphere was used to facilitate the combustion of the grafted organic compound. As expected, a more important mass loss was obtained under air, showing that the organic matter departure was partial under nitrogen atmosphere (Supplemental data, Figure S2). From the overall mass loss, the amount of grafted NI was estimated to be 0.44 mol/mol of kaolinite structural formula (Al_2_O_3_Si_2_O_2_(OH)_4_).

#### 2.1.3. ^13^C NMR

**Figure 3:**
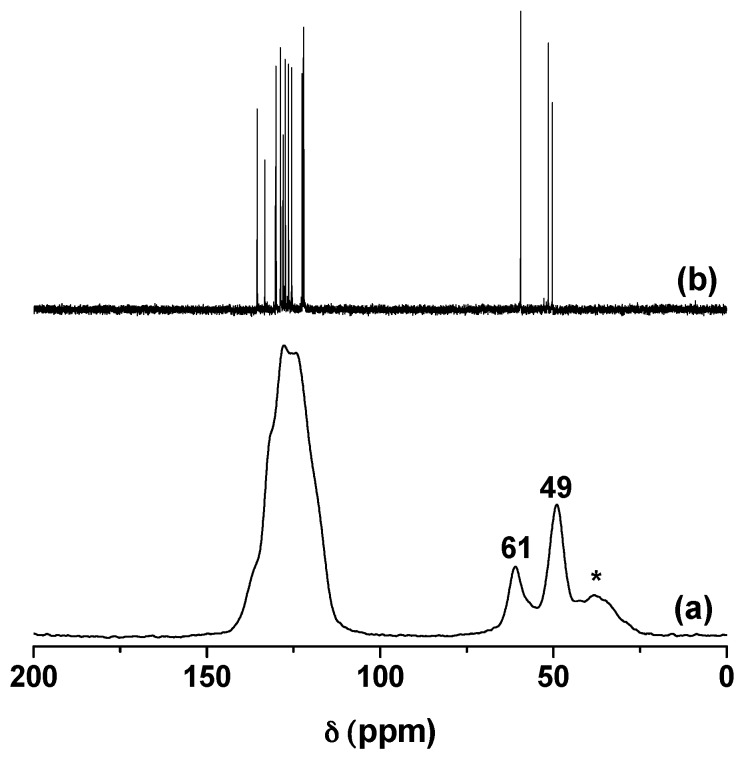
(**a**) Solid state ^13^C CP/MAS NMR spectrum of K-NI and; (**b**) liquid phase ^13^C NMR spectrum of NI in D_2_O as solvent. Spinning side band is marked by *.

^13^C NMR is the appropriate tool for determining the structure of the grafted compound, and thus ensuring that there were no structural changes of the organic cation after grafting. Figure 3a shows the ^13^C CP/MASNMR spectrum of K-NI. The broad peak between 143 ppm and 110 ppm corresponds to the aromatic carbons of the naphthyl group and of the imidazolium ring. The peak at 61 ppm corresponds to the carbon of the Al–O–**C** bond. It shows a slight chemical shift difference with the one of the equivalent carbon in the liquid phase (59.4 ppm, Figure 3b). The signals of the two aliphatic carbons linked to the imidazolium nitrogen atoms overlap in a peak centered at 49 ppm. The broad peak centered at 37 ppm is a spinning side band of the aromatic peaks. The ^13^C NMR spectra of NI in the liquid phase and the one of K-NI are almost superimposable regardless of the slight chemical shifts observed for the carbons close to the Al–O–C bond resulting from the grafting reaction. This clearly demonstrates that NI did not undergo any structural modification in the interlayer space of kaolinite, except for the reaction of the hydroxyl group with the kaolinite aluminol groups.

#### 2.1.4. FTIR

**Figure 4:**
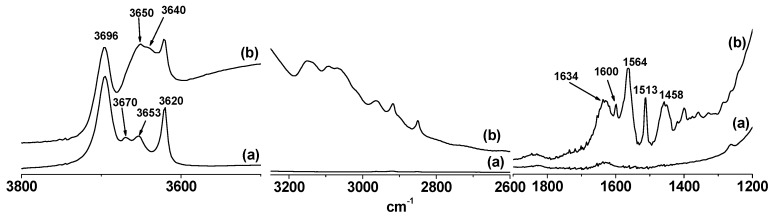
FTIR Spectra of (**a**) kaolinite and (**b**) K-NI.

K-NI was also characterized by IR spectroscopy. Magnifications were performed on three relevant parts of the spectrum (3800–3500 cm^−1^, 3250–2600 cm^−1^ and 1900–1200 cm^−1^). They are presented in Figure 4 (the full spectra are presented in Supplemental data, Figure S3). In the O–H stretching region of kaolinite, the well-known four bands of kaolinite appear at 3696 cm^−1^, 3670 cm^−1^, 3653 cm^−1^, and 3620 cm^−1^, accounting for the stretching vibration modes of interlayer and internal OH [30]. On K-NI, the disappearance of the band at 3670 cm^−1^ was observed, while the one at 3653 cm^−1^ shifted slightly to 3650 cm^−1^. A new band with low intensity also appeared at 3640 cm^−1^. The bands at 3696 cm^−1^ and 3620 cm^−1^ remained unchanged. These observations confirm the perturbation of the OH vibrational modes of kaolinite by the grafted ionic liquid. Between 3190 cm^−1^ and 3000 cm^−1^, the stretching bands of the aromatic C–H of the naphthyl and imidazolium groups were observed. The C–H stretching bands of aliphatic carbons were present between 3000 and 2830 cm^−1^. These observations confirm the presence of the ionic liquid in the interlayer space of kaolinite, as showed by XRD, TGA and ^13^C NMR characterization.

The band related to the bending vibrational mode of physisorbed water molecules was observed at 1630 cm^−1^. This band appeared on the spectrum of kaolinite, but with a low intensity, indicating that the chemical modification increased the hydrophilicity of kaolinite as can be seen also by the TGA results. A similar increase of the hydrophilicity of kaolinite after modification by ionic liquids was reported in the literature [21,22,31]. The broad band centered at 3490 cm^−1^ (Figure 4b and Supplemental data, Figure S3) also accounts for hydrogen bonded water molecules. Various C–C stretching bands of the naphthyl group and of the imidazolium ring were also observed on K-NI (1600 cm^−1^, 1564 cm^−1^, 1513 cm^−1^ and 1458 cm^−1^).

These characterizations demonstrate the grafting of NI on the interlayer aluminolsurfaces of kaolinite. The counter-anion, which maintains the electrical neutrality of the material, was then available for exchange with other anions. The mobility of the counter-anion was previously used for the electroanalysis of anions, with the limitation that large anions could not fit into the confined interlayer space when the grafted organic cation was not large enough [21].

### 2.2. Application

#### 2.2.1. Ferricyanide Electroanalysis

**Figure 5:**
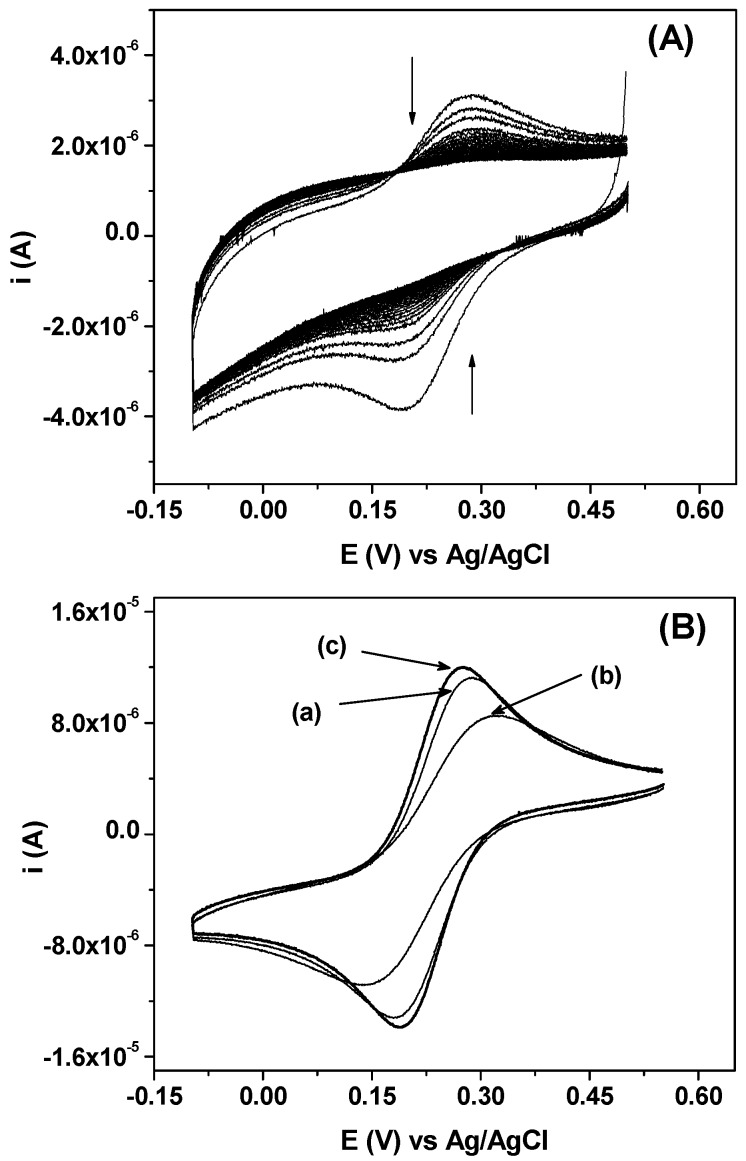
(**A**) Series of 30 consecutive cyclic voltammograms recorded on glassy carbon electrode (GCE)/K-NI in 0.2 M KCl at 100 mV s^−1^ after 10 min pre-concentration in 10^−4^ M K_3_Fe(CN)_6_; (**B**) Cyclic voltammograms recorded at 100 mV s^−1^ in a 1 mM solution of K_3_Fe(CN)_6_, and 0.2 M KCl as supporting electrolyte on: (a) GCE; (b) GCE/K and (c) GCE/K-NI.

Ferricyanide is a bulky anion with spherical shape, having a diameter of about 8.8 Å. It provides a well-defined and reversible one electron system (Fe(CN)_6_^3−/4−^) on various electrodes. Despite its size, this anion should be able to penetrate into the large interlayer space of K-NI (10.8 Å). To confirm the ability of K-NI to accumulate ferricyanide, a glassy carbon electrode (GCE) modified by a thin film of K-NI (GCE/K-NI) was dipped in a 0.1 mM potassium ferricyanide solution for 10 min, fully rinsed with deionized water and then transferred in a 0.2 M KCl solution. Cyclic voltammograms were then immediately recorded (Figure 5A). During the first scan, the well-defined oxidation/reduction signal of the system was observed. This signal gradually decreased with the number of cycles and became flat after about 25 cycles. This shows that during the pre-concentration step, chlorides have been effectively replaced by ferricyanide, a process driven by a concentration gradient, promoting the intercalation. In the detection medium, the opposite arises: Chloride migration was favoured by the concentration gradient, and ferricyanide was transferred back into the bulk solution. Ferricyanide anions were poorly retained by the modified kaolinite contrarily to what has been observed by some authors by using a chitosan or amine functionalized montmorillonite modified electrodes [32,33]. The ease of release observed shows that K-NI modified electrode could be used as a sensor for the quantification of ferricyanides, and certainly other electroactive anions.

Figure 5B shows the signals of the electroactivity of Fe(CN)_6_^3−^ recorded in identical experimental conditions, respectively, on bare glassy carbon electrode (GCE), kaolinite modified glassy carbon electrode (GCE/K) and K-NI modified glassy carbon electrode (GCE/K-NI), in the electrolytic solution containing ferricyanide 1 mM and KCl 0.2 M. For these electrodes, almost no variation on signal intensities was observed for multiple scans, thus, the voltammograms presented are the first scans. The signal obtained at GCE/K-NI was slightly more intense as compared to the one recorded on GCE. The gain in intensity reflects the affinity of the film for Fe(CN)_6_^3−^ and confirms that this anion can be intercalated into the modified interlayer space of kaolinite [21]. On GCE/K, there was a significant decrease in current intensities, a result expected, considering the poor anion exchange capacity of pristine kaolinite [34,35,36,37]. The signal observed was mainly due to the irregularity of the film at the surface of the electrode. The cyclic voltammograms also showed that the peak potential separation (ΔE) is important in the case of GCE/K (175 mV), as compared to GCE (99 mV), another indication that the diffusion of Fe(CN)_6_^3−^ through the kaolinite film is not favoured. For GCE/K-NI, the value of ΔE was less important (81 mV) when compared to the value obtained on GCE, confirming once again that K-NI displays a strong affinity to ferricyanide anions. In a previous publication, using kaolinite modified by an ionic liquid with a *d*-value of 15.8 Å, the ferricyanide electrochemical signal was found to be poorly defined, as compared to that recorded on GCE. This result was explained by the relatively small *d*-value that did not allow the intercalation of the bulky ferricyanide anions [21]. This result confirms that for these kaolinite nanohybrid materials, the *d*-value strongly controls the electrochemical signal during the electroanalysis of anions.

Series of voltammograms were recorded on GCE/K-NI, varying the scan rate from 10 to 1000 mV (Figure S4). It was found that the peak current increased with the scanning rate. Plotting the peak currents as a function of the square root of the scanning rate resulted in a straight line (Inset Figure S4), demonstrating that the process at the electrode surface was diffusion controlled [33,38].

#### 2.2.2. P-Nitrophenol (PNP) Adsorption

The ability of K-NI to pre-concentrate anions has been used to adsorb PNP, an organic compound widely used in the chemical industry as an intermediate for the synthesis of various compounds. The presence of PNP in industrial effluents causes significant environmental issues. Therefore, the development of PNP removal methods is of interest [39,40,41]. PNP is a weak acid with a pKa of 7.1. In water, it undergoes partial ionization to form the nitrophenolate anion. This flat anion could fit between the layers of K-NI to replace chloride. Preliminary experiments showed that no quantitative adsorption could be obtained on pristine kaolinite (Supplementary data, Figure S5A. There was a quantitative accumulation of PNP when K-NI was used as adsorbent in water (Figure S5A). K-NI probably acts as a sieve that selectively extracts the nitrophenolate ions from the solution. Only the anionic form seemed to be able to be accumulated quantitatively as almost no adsorption was observed when ethanol was used as solvent (Supplementary data, Figure S5B).

**Figure 6:**
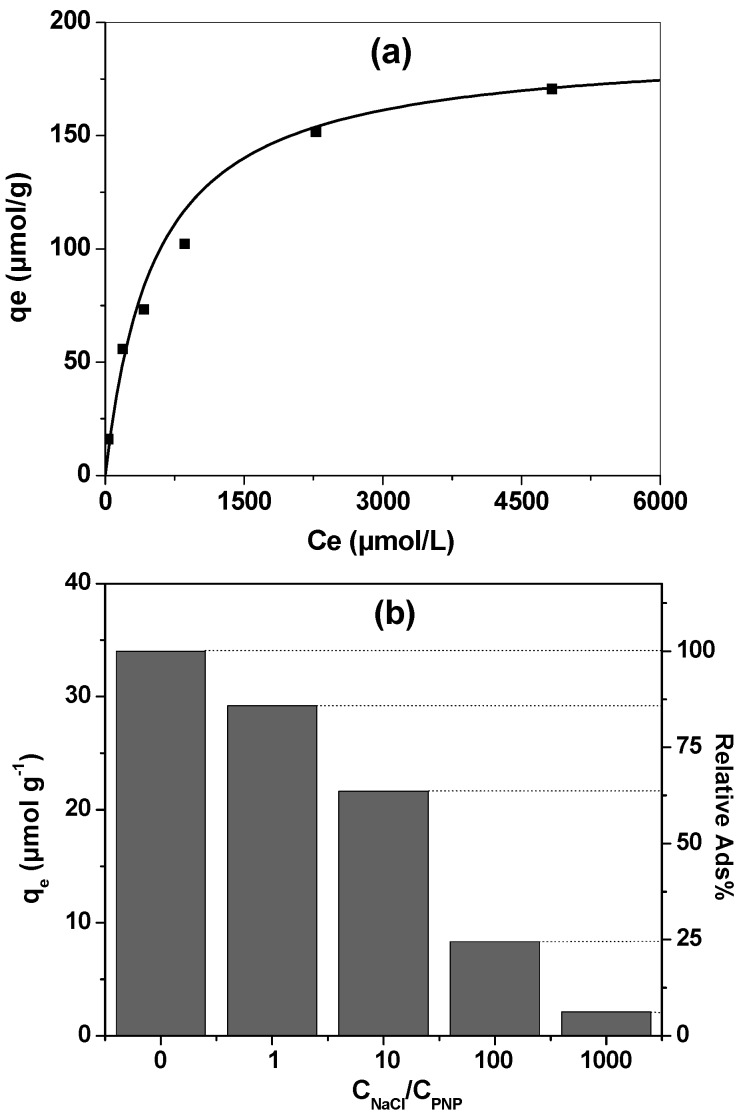
PNP adsorption on K-NI 1 g L^−1^: (**a**) adsorption isotherm obtained at room temperature. The curve is calculated following a Langmuir isotherm model; (**b**) variation of the adsorption capacity of K-NI depending on the concentration of NaCl, C_PNP_ = 10^−4^ M. The relative adsorption% was determined using the adsorption capacity for C_NaCl_ = 0 Mas reference.

Figure 6a depicts the variation of the adsorption capacity of K-NI when varying the initial concentration of PNP. There was a gradual increase at low concentrations, reflecting the favoured exchange rate of chloride by nitrophenolate. At higher concentrations, the increase in adsorption capacities becomes less important. The shape of these experimental data is similar to a Langmuir-type isotherm. This was confirmed by applying the Langmuir model to the experimental data with a good correlation (R^2^ > 0.99). The maximum adsorption capacity obtained from this model was 190 µmol/g, six times less important than the calculated anion exchange capacity of the material (1127 µmol/g) as estimated by TGA. This difference could be explained by the steric hindrance due to the bulky grafted NI group. Indeed, when using a kaolinite-based nanohybrid material functionalized with less bulky ionic liquids, chlorides were completely exchanged by large organic anions such as salicylates [31].

The aromatic ring of PNP could provide strong π–π interactions between this compound and NI, taking advantage of the presence of the naphthyl groups and the imidazolium ring. These interactions could lead to a preferential accumulation of this compound through stabilization in the interlayer space due to π–π interactions. To investigate this hypothesis, the adsorption of PNP at 10^−4^ M was performed in a solution containing NaCl at various concentrations, ranging from 10^−4^ to 10^−1^ M. The results are presented in Figure 6b. When increasing the NaCl concentration, the ability of K-NI to accumulate PNP decreased. However, if the effect of the presence of chlorides on the process is quantified, for equivalent amounts of PNP and Cl^−^ in solution, the adsorption capacity decreased only by 14%. When there were 10 times, 100 times, or 1000 times more chlorides, the decrease was 36%, 75%, and 85%, respectively. If the affinity of the cationic interlayer pillars for these two anions were similar, the adsorption of PNP would be more strongly reduced. In addition, PNP was supposed to be less favoured because of steric hindrance. These observations demonstrate that there is an affinity for PNP adsorption in the interlayer space of K-NI, favoured by the presence of the aromatic ring, and/or by the hydrogen bonds that could be formed between the aluminol surface and nitrophenolates anions.

## 3. Materials and Methods

### 3.1. Chemicals

1-(Chloromethyl) naphthalene (≥97%), 1-(2-hydroxyethyl)-imidazole (97%), p-nitrophenol (98%), and potassium ferricyanide were obtained from Aldrich (Aldrich, St. Louis, MO, USA). All other chemicals (Potassium chloride, dimethylsulfoxide, anhydrous ethyl ether, and methylene dichloride) were of analytical grade.

### 3.2. Synthesis of Ionic Liquids

1-methylnaphthyl-3-(2-hydroxyethyl) imidazolium chloride (NI) (Scheme 1) was prepared by dissolving 27 mmol of 1-(2-hydroxyethyl)-imidazole in 10 mL of dichloromethane. 27 mmol of 1-(Chloromethyl) naphthalene were then added, and the mixture refluxed at 80 °C for five days at constant stirring. The yellow gold ionic liquid obtain was highly viscous at room temperature (20 °C). ^1^H NMR: δ 3.6 ppm (m, 2 H, HO-CH_2_-C**H_2_**-N-), 3.9 ppm (m, 2 H, HO-C**H_2_**CH_2_-N-), 5.3 ppm (s, 2 H, C_10_H_7_-C**H_2_**-N^+^-), 6.9–7.5 (m, 9 H, -N-C**H**-C**H**-N^+^- and C_10_**H_7_**-CH_2_-N^+^-), 8.5 ppm (s, 1 H, N-C**H**-N^+^-), ^13^C NMR: δ 50.3 ppm (1 C, HO-CH_2_-**C**H**_2_**-N-), δ 51.4 ppm (1 C, -N-CH-CH-N^+^-**C**H_2_-C-C_10_H_7_), 59.4 ppm (1 C, HO-**C**H_2_-CH**_2_**-N-), 122.0–135.5 ppm (13 C, -**C**H-N-**C**H-**C**H-N^+^-CH_2_-**C_10_**H_7_-).

### 3.3. Preparation of Kaolinite and Nanohybrid Materials

Well crystallized kaolinite (KGa-1b, Georgia) was obtained from the Source Clays Repository of the Clay Minerals Society (Purdue University, West Lafayette, IN, USA). Purification of KGa-1b and intercalation of dimethylsulfoxide (DMSO) to obtain the K-DMSO pre-intercalate was achieved by following previously described procedures [23,24]. For the grafting of NI, 0.5 g of K-DMSO was dispersed in 2 g of the ionic liquid. The mixture was stirred under nitrogen for 4 h, with the temperature being gradually increased to 180 °C. The resulting mixture was fully washed three times with isopropanol to remove the excess of ionic liquids and dried in oven at 80 °C for 5 h. The material was then stirred for 48 h in 100 mL of distilled water to remove the ungrafted compounds trapped in the interlayer space. The grafted kaolinite (K-NI) was recovered by centrifugation and dried in an oven at 70 °C for 5 h.

### 3.4. Electrochemistry

Thin film electrodes were used for electrochemical experiments, by coating kaolinite or K-NI on the surface of a glassy carbon electrode (GCE) by using a drop coating strategy. Practically, 5 µL of a suspension of clay in deionised water (4 g L^−1^) was carefully deposited on GCE and dried in open air for about 45 min. The thin film electrode was used as a working electrode, a platinum grid as a counter electrode, and an Ag/AgCl (KCl saturated) electrode as a reference. All of the potential values in this work are referred to this reference electrode. Cyclic voltammetry was used for all electrochemical experimentations using a VersaSTAT 3 potentiostat (Princeton Applied Research, Oak Ridge, TN, USA) controlled by V3-studio software (Ametek, Berwyn, PA, USA).

### 3.5. p-Nitrophenol (PNP) Adsorption

In a typical PNP adsorption experiment, 3 mg of K-NI was dispersed in a 3 mL PNP solution and stirred for 10 h. After centrifugation, the UV-Vis spectrum of the supernatant was recorded on a GENESYS 10S Vis Thermo Scientific spectrophotometer (Thermo Scientific, Waltham, MA, USA), after adding one drop of NaOH 1 M to convert the PNP to the nitrophenolate form. The residual concentration was determined by using the absorbance at 400 nm. The same procedure was used to study the interference of chloride, with the controlled amount of this anion added in the solution before adsorption experiments.

### 3.6. Characterization

Powder XRD patterns were recorded on a Philips PW 3710 diffractometer (Philips Analytical, Amsterdam, Netherlands) operating with Cu-Kα radiation (λ = 1.54056 Å) using a generator with a voltage of 45 kV and a current of 40 mA.

Thermal gravimetric analyses (TGA) were recorded using a TA instrument Q5000 (TA Instrument, New Castle, DE, USA) under nitrogen flow (100 mL/min) or house air flow (100 mL/min) at a heating rate of 10 °C/min.

^1^H and ^13^C NMR spectra in solution were recorded using a Bruker 400 MHz spectrometer (Bruker, Billerica, MA, USA), with deuterated water as solvent. Solid-state ^13^C NMR CP/MAS spectrum was collected on a Bruker AVANCE 200 spectrometer (Bruker, Billerica, MA, USA) spinning at 4500 Hz.

KBr pellets were prepared for the IR analysis and the spectrum recorded on a Thermo scientific Nicolet 6700 FT-IR equipment (Thermo scientific, Waltham, MA, USA).

## 4. Conclusions

This work confirms that the grafting of increasingly complex organic compounds on the inner surfaces of kaolinite becomes well mastered, which allows the design of systems in which the grafted cations can exchange and differentiate anions according to their size and their chemical properties. The next steps should be the control of the functionality degree of these nanohybridmaterials to avoid steric hindrance that drastically reduces their performances. Once grafted, the organic cations of intercalated ionic liquids set rigidly the interlayer distance which can consequently be designed and controlled by the careful choice of the size and shape of the organic cation. A necessary further step will be to control equally the distances between cations which will result in controlled microporosity. The controlled grafting in the interlayer spaces of kaolinite with higher d-spacing will also probably complete in the near future the goal of using this confined space as a non-centrosymmetric nanoreactor.

## Supplementary Materials

The following are available online at www.mdpi.com/1996-1944/10/9/1006/s1, Figure S1: Powdered XRD patterns of (a) kaolinite, (b) K-NI and K-NI sonicated for 30 min in (c) water and (d) toluene, Figure S2: TGA curves of K-NI (a) under N_2_, (b) under air, Figure S3: FTIR Spectra of (a) kaolinite and (b) K-NI, Figure S4: Effect of the scanning rate on the signal recorded on GCE/K-NI. Voltammograms recorded in 1 mM solution of K_3_Fe(CN)_6_ and 0.2 M KCl as supporting electrolyte at varying scanning rate. Inset variation of peak currents as a function of the square root of the scan rate, Figure S5: UV-Vis spectra of (A) 10^−4^ M PNP aqueous solution: (a) before adsorption, after 10 h contact time with (b) kaolinite 2 g L^−1^ and (c) K-NI 2 g L^−1^; (B) 5 × 10^−5^ M PNP ethanol solution, (a) before adsorption and (b) after 10 h contact time with K-NI 1 g L^−1^.
