# Supplementary material for: Characterization and Applications of Kaolinite Robustly Grafted by an Ionic Liquid with Naphthyl Functionality
Source: Materials (Basel). 2017 Aug 29;10(9):1006. doi: 10.3390/ma10091006 (PMC5615661; doi:10.3390/ma10091006)
Supplement: Supplementary file 1 [file materials-10-01006-s001.pdf]

Supplementary Materials

Characterization and applications of robustly grafted kaolinite using ionic liquid with naphthyl functionality

Gustave Kenne Dedzo[1,2]* and, Christian Detellier[1]

[1] Center for Catalysis Research and Innovation and Department of Chemistry and Biomolecular Sciences, University of Ottawa, Ottawa, Ontario K1N 6N5, Canada.
[2] Laboratory of Analytical Chemistry, Faculty of Science, University of Yaounde I, B.P. 812, Yaoundé, Cameroon.
* Correspondence: gkennede@uottawa.ca

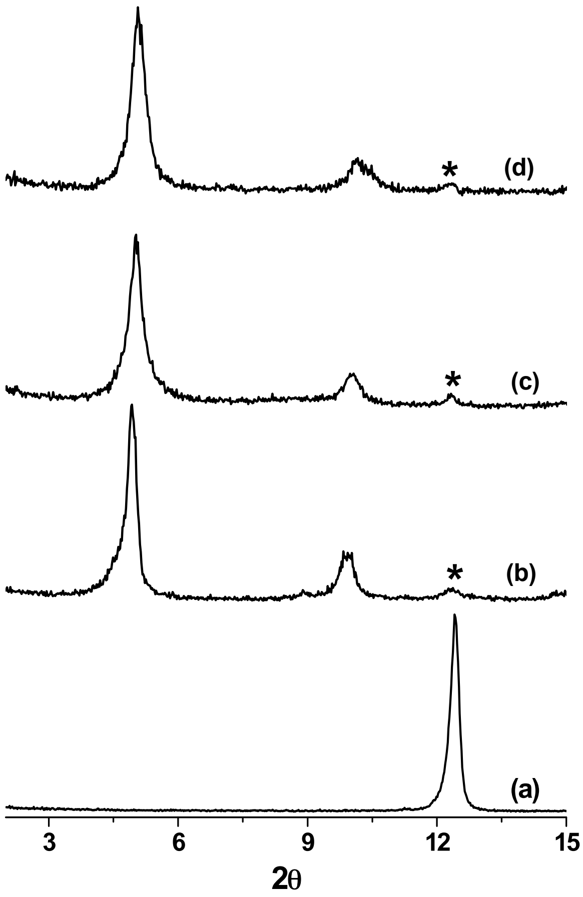

Figure S1. Powdered XRD patterns of (a) kaolinite, (b) K-NI and K-NI sonicated for 30 minutes in (c) water and (d) toluene.

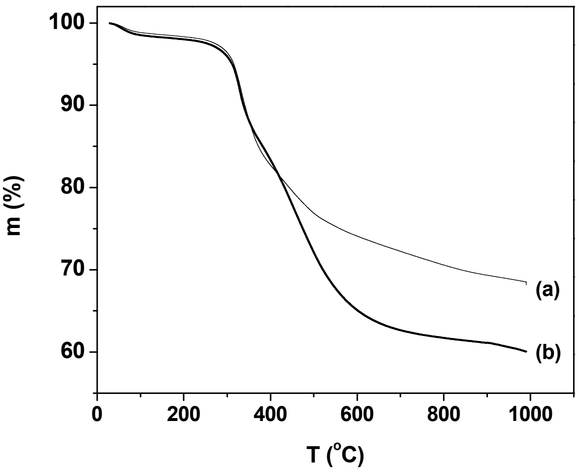

Figure S2.TGA curves of K-NI (a) under N_2, (b) under air.

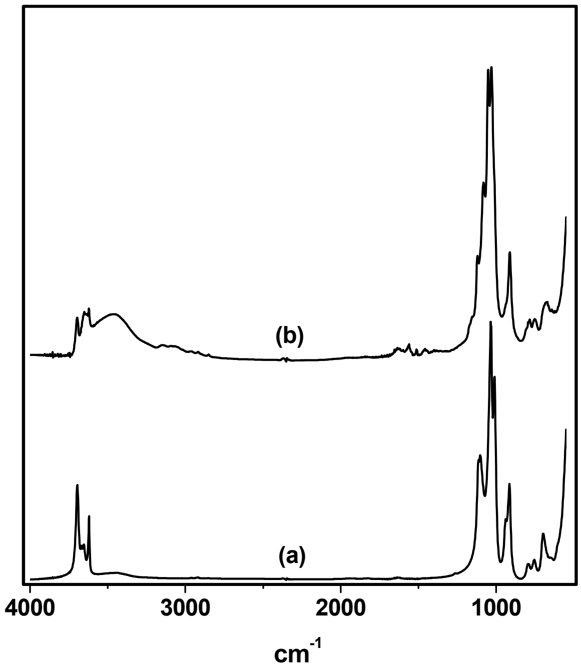

Figure S3. FTIR Spectra of (a) kaolinite and (b) K-NI.

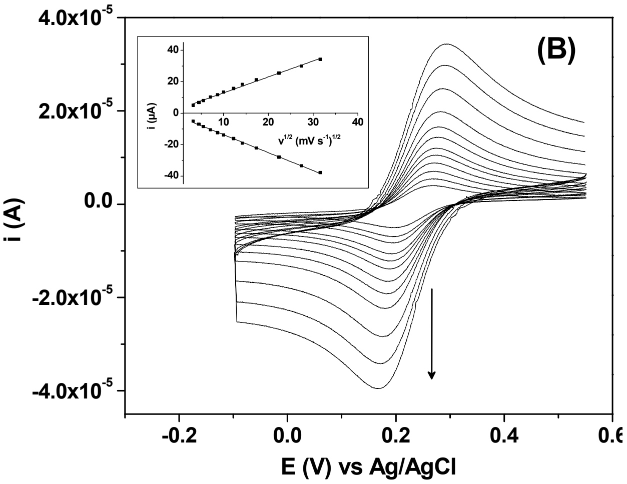

Figure S4. Effect of the scanning rate on the signal recorded on GCE/K-NI. Voltammograms recorded in 1 mM solution of $K_3Fe(CN)_6$ and 0.2 M KCl as supporting electrolyte at varying scanning rate. Inset variation of peak currents as a function of the square root of the scan rate.

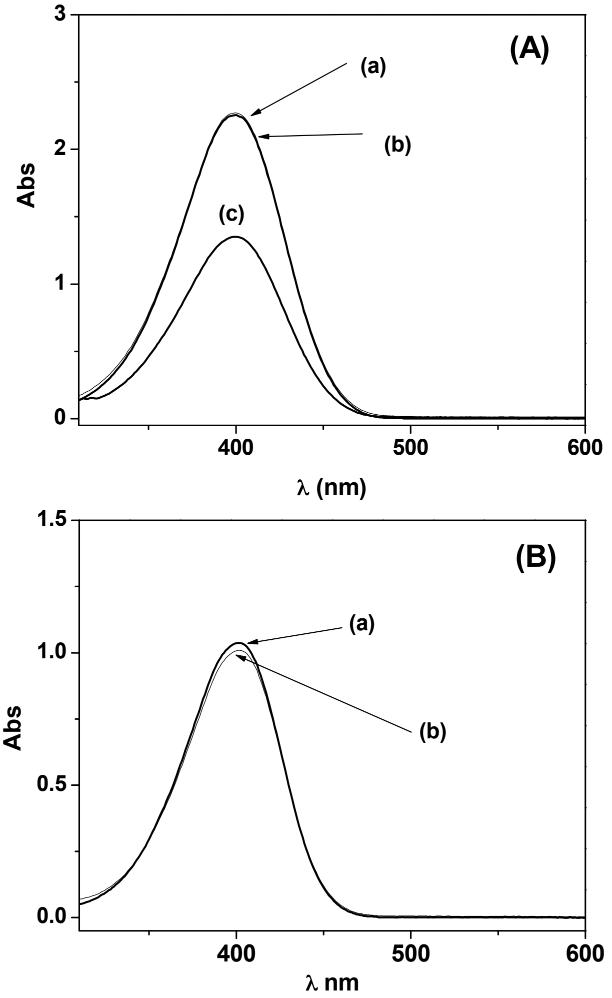

Figure S5: UV-Vis spectra of (A) 10^{-4} M PNP aqueous solution: (a) before adsorption, after 10 hours contact time with (b) kaolinite 2g $L^{-1}$and (c) K-NI 2g L^{-1}; (B) $5x10^{-5}$ M PNP ethanol solution, (a) before adsorption and (b) after 10 hours contact time with K-NI 1g L^{-1}.
